# Comparative evaluation of dry and liquid RIME LAMP in detecting trypanosomes in dead tsetse flies

**DOI:** 10.4102/ojvr.v85i1.1543

**Published:** 2018-10-03

**Authors:** Peter Nambala, Janelisa Musaya, Kyoko Hayashida, Emmanuel Maganga, Edward Senga, Kelita Kamoto, John Chisi, Chihiro Sugimoto

**Affiliations:** 1 Department of Basic Medical Sciences, University of Malawi, Malawi; 2 Department of Pathology, University of Malawi, Malawi; 3 Research Centre for Zoonosis Control, Hokkaido University, Japan; 4 Mikolongwe Veterinary College of Agriculture and Food Security, Limbe, Malawi

## Abstract

Xenomonitoring is an important approach in assessing the progress of trypanosomiasis control as well as in estimating the endemicity of trypanosomes in affected areas. One of the major challenges in this approach is the unavailability of sensitive and easy to use xenomonitoring tools that can be used in the remote areas where the disease occurs. One tool that has been used successfully in detecting the parasites in tsetse flies is the repetitive insertion mobile element loop-mediated isothermal amplification (RIME LAMP). This tool has recently been modified from the liquid form to dry form for use in remote areas; however, uptake for use in the field has been slow. Field-collected tsetse flies were used to evaluate the performance of dry RIME LAMP over the conventional liquid RIME LAMP. All the samples were also subjected to internal transcribed spacer 1 (ITS1) ribosomal deoxyribonucleic acid (DNA) polymerase chain reaction (PCR) as a standard. ITS1-PCR-positive samples were further sequenced for confirmation of the species. A total of 86 wild tsetse flies were left to dry at room temperature for 3 months and DNA was extracted subsequently. All 86 flies were *Glossina morsitans morsitans*. From these, dry RIME LAMP detected 16.3% while liquid RIME LAMP detected 11.6% as infected with trypanosomes. Ten positive samples on ITS1-PCR were sequenced and all were shown to be trypanosomes. The use of dry RIME LAMP in the field for xenomonitoring of trypanosomes in tsetse flies will greatly contribute towards control of this neglected tropical disease as it provides the cheapest, fastest and simplest way to estimate possible human infective trypanosome infection rates in the tsetse fly vectors.

## Introduction

Human African trypanosomiasis (HAT) is an important public health problem that affects rural populations of 36 sub-Saharan African countries (Franco et al. 2014). The disease is caused by trypanosomes belonging to the subgenus *Trypanozoon* (Bruce 1915). *Trypanosoma brucei rhodesiense* causes an acute disease in eastern, central and southern parts of Africa, while *Trypanosoma brucei gambiense* causes chronic diseases and occurs in west and central Africa (Brun & Blum 2012). Other parasites of the subgenus *Trypanozoon* are *Trypanosoma brucei brucei, Trypanosoma evansi* and *Trypanosoma equiperdum*, which cause Nagana, Surra and Dourine in livestock, respectively. The epidemiology of sleeping sickness follows the parasite–vector interactions within a particular environment (Dale et al. 1995); therefore, the risk of host infection is through a tsetse fly bite. In Malawi, it has been shown that tsetse flies are infected with the human infective *T. b. rhodesiense* (Musaya et al. 2017). Therefore, deploying control measures in areas infested with infected tsetse flies would be ideal in reducing the prevalence of the disease. In limited-resource settings, tools that are sensitive and easy to use will be very useful in the surveillance of trypanosome infection rate in tsetse flies.

In any particular environment, success of trypanosomiasis control is dependent upon the use of appropriate diagnostic tools. Detection of trypanosomes in vectors for surveillance has previously depended on dissection of tsetse flies for mid-guts and salivary parts for microscopy and polymerase chain reaction (PCR) for amplification of trypanosome deoxyribonucleic acid (DNA) (Basheer et al. 2013; Malele et al. 2011). These techniques are labour intensive and not conducive for control programmes.

Not all tsetse flies positive for trypanosome DNA are indicative of an established infection as some trypanosome DNA may originate from the blood meal that the flies had taken prior to analysis (Macleod et al. 2007). However, for control purposes any tsetse fly infection is important for deployment of control strategy instead of waiting until infection is established in humans or animals. Despite technological advancement in the diagnosis of trypanosomes, the uptake of these techniques has been minimal and many diagnostic settings continue to rely on insensitive and unsatisfactory parasitological or serodiagnostic techniques (Njiokou et al. 2004; Ouma et al. 2000)

A more sensitive diagnostic and surveillance technique called repetitive insertion mobile element loop-mediated isothermal amplification (RIME LAMP) has been developed and evaluated for detection of trypanosomes using tsetse fly mid-guts (Alibu et al. 2015; Njiru et al. 2008a). However, this test still poses a challenge in tropical and developing countries where the disease is endemic as the reagents and field samples still require expensive equipment, which are unfortunately not available in most places. A technique for use in surveillance and control programmes should be able to detect trypanosomes in a variety of specimens that can be easily collected and stored with very minimal logistical requirements.

Dry RIME LAMP for detection of HAT has been developed through modification of the conventional liquid RIME LAMP (Hayashida et al. 2015). The unique feature in the components of dry RIME LAMP is that all reaction reagents are air-dried on a lid of individual reaction tubes for easy storage, transportation and use at room temperatures unlike liquid RIME LAMP, which requires cold chain for storage of reagent components. Dry RIME LAMP was demonstrated to have robust performance in detecting HAT in artificially parasite mixed blood (Hayashida et al. 2015). However, use of dry RIME LAMP for detection of trypanosomes in field-collected samples has not been demonstrated. Detection of trypanosomes in tsetse flies may accelerate the critical advances in the control of both HAT and animal African trypanosomiasis (AAT). In this study, we demonstrate that dry RIME LAMP has the potential to be used as a surveillance tool to detect trypanosomes in field-caught tsetse flies. The study was carried out in the Liwonde Wildlife Reserve, Machinga District, Malawi.

## Materials and methods

### Study area

The fieldwork was conducted in the Liwonde Wildlife Reserve, Machinga District, Malawi. This is located in the eastern part of Malawi, and being a protected area, it is rich in wildlife. Wildlife provides the main source of blood meals for tsetse flies. The main feature is the Shire river, which flows along the wildlife reserve and forms a valley. On the highlands east of this area, land use is characterised by mixed-crop agriculture, livestock and higher human population densities. A game fence, which is irregularly maintained, physically marks the boundary between the protected and settlement areas.

#### Tsetse collection

Tsetse flies were collected using stationary Epsilon traps that were placed near the main camp for 24 hours. Fly round was also used to cover an area of about 5 kilometres in the same location where traps were set. Fly rounds involved the use of a blue or black screen tent mounted in the body of a car that was baited with tsetse fly odour attractants (3-n propyl phenol, 1-octen-3-ol, 4-methyl phenol). Collected dead and live tsetse flies were dried at room temperature and individually put in single Eppendorf tubes containing desiccants. Cotton wool was placed in the middle of the tubes to avoid contact of the desiccants with the tsetse fly. The flies were stored at room temperature for a period of 3 months before analysis.

#### Isolation of parasite deoxyribonucleic acid

Dry whole tsetse flies were transferred to new Eppendorf tubes and crushed using a sterilised plastic pestle. Parasite DNA was extracted from the crushed tsetse flies using Roche DNA isolation kit (Mannheim, Germany) as per the manufacturer’s instructions. The DNA was suspended in 100 *µ*L of PCR water and stored at -20 °C.

#### Amplification of trypanosome deoxyribonucleic acid using repetitive insertion mobile element loop-mediated isothermal amplification

**FIGURE 1:**
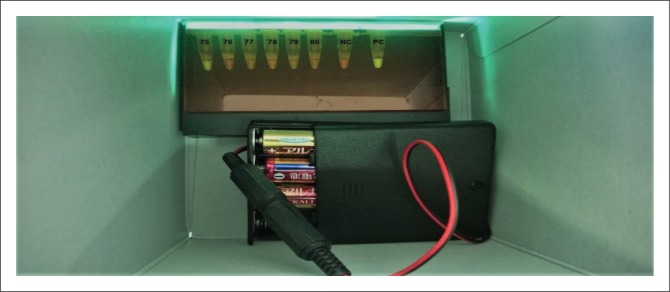
Visualisation of dry repetitive insertion mobile element loop-mediated isothermal amplification using battery-operated LED light. Positive and negative samples turn green and orange, respectively, under LED light.

Dry RIME LAMP was used to amplify the parasite DNA. Liquid RIME LAMP was also used alongside the dry RIME LAMP. Six LAMP primers (Table 1) were used to detect the *Trypanozoon* group of the trypanosome parasite for both dry and liquid RIME LAMP (Hayashida et al. 2015; Njiru et al. 2008a; Njiru et al. 2008b). For dry RIME LAMP, all LAMP reagents were placed on the lid of each 0.2-mL microtube and air-dried as described by Hayashida et al. (2015). The purified genomic DNA from the dead tsetse fly was used as a reaction template, 2 *μ*L of DNA plus 1 *μ*L of 25X LAMP buffer (500 mM Tris–HCl, 250 mM KCl and 100 mM MgSO_4_), 1.5 *μ*L of 100 mM MgSO_4_, and 0.1% Triton X-100 in nuclease-free water were added to make a 25-*μ*L reaction mix. The tubes were turned upside down and mixed well so that the dried reagents were completely reconstituted. The tubes were incubated at 65 °C for 60 min and the amplification reactions were visualised using a battery-operated light-emitting diode (LED) illuminator (Figure 1).

**TABLE 1:** Nucleotide sequences of internal transcribed spacer 1-polymerase chain reaction and repetitive insertion mobile element loop-mediated isothermal amplification primers.

Primer	Sequence (5′–3′)	Length
ITS1 CF	CCGGAAGTTCACCGATATTG	20
ITS1 BR	TTGCTGCGTTCTTCAACGAA	20
SRA B537	CCATGGCCTTTGACGAAGAGCCCG	24
SRA B538	CTCGAGTTTGCTTTTCTGTATTTTTCCC	28
RIME F3	CTGTCCGGTGATGTGGAAC	19
RIME B3	CGTGCCTTCGTGAGAGTTTC	20
RIME FIP	GGAATACAGCAGATGGGGCGAGGCCAATTGGCATCTTTGGGA	42
RIME BIP	AAGGGAGACTCTGCCACAGTCGTCAGCCATCACCGTAGAGC	41
RIME LF	GCCTCCCACCCTGGACTC	18
RIME LBm	CCAGACCGATAGCATCTCAG	18
SRA F3m	AACAAGTATCGGCAGCAACC	18
SRA B3	TCTTACCTTGTGACGCCTG	19
SRA FIPm	CTGCGTTGAGTACGCATCTTGCACAGACCACAGCAACATC	40
SRA BIP	CGCTCTTACAAGTCTTGCGCCCTTCTGAGATGTGCCCACTG	41
SRA LFm	CGGCATAAAGCGCTGAGA	19
SRA LB	GCAGCGACCAACGGAGCC	-

ITS1, internal transcribed spacer 1; RIME, repetitive insertion mobile element; SRA, serum-resistant associated gene.

For the liquid RIME LAMP, amplification was performed using 25 *μ*L total volume for one reaction: 2 *µ*L of template DNA, 1 *μ*L of 25X LAMP buffer (500 mM Tris–HCl, 250 mM KCl and 100 mM MgSO_4_), 1.5 *μ*L of 100 mM MgSO_4_, 1.4 *μ*L of 25 mM dNTPs, 1 *μ*L of each primer, 1 *μ*L of *Bst* 2.0 DNA polymerase (New England Biolabs, Japan), 2 *μ*L of 2 M Trehalose, 1 *μ*L of colori-fluorometric indicator (CFI; 3 mM hydroxy-naphthol blue (MP Biomedicals, Aurora, OH, USA) and 0.35% v/v GelGreen (10,000X solution; Biotium, Hayward, CA, USA) and volume adjusted with nuclease-free water containing 0.1% Triton X-100. The amplification was monitored on the FAM channel using a real-time PCR machine (CFX96; Bio Rad, Japan) at 62 °C for 60 min.

#### Amplification of trypanosome deoxyribonucleic acid using serum-resistant associated gene loop-mediated isothermal amplification

All samples were also subjected to serum-resistant associated gene (SRA) LAMP that is specific for the detection of the SRA responsible for infectivity of *T. b. rhodesiense* to humans. Six primers used for SRA LAMP are also shown in Table 1 (Hayashida et al. 2015). Amplification was performed using 25-*μ*L total volume for one reaction: 2 *μ*L of template DNA, 1 *μ*L of 25X LAMP buffer (500 mM Tris–HCl, 250 mM KCl and 100 mM MgSO_4_), 1.5 *μ*L of 100 mM MgSO_4_, 1.4 *μ*L of 25 mM dNTPs, 1 *μ*L of each primer, 1 *μ*L of *Bst* 2.0 DNA polymerase (New England Biolabs), 2 *μ*L of 2 M Trehalose, 1 *μ*L of CFI and volume adjusted with nuclease-free water containing 0.1% Triton X-100. The amplification was monitored in a real-time PCR machine at 62 °C for 60 min.

#### Visualisation of dry repetitive insertion mobile element loop-mediated isothermal amplification reaction

Colori-fluorometric indicator dye that contains GelGreen was included in the dry tube to enable direct visualisation of amplified DNA products on a battery-operated handheld LED illuminator that emits 500 nm wavelength of light (Figure 1). Amplified DNA was detected by direct visual inspection of colour change of CFI dye under LED light. Positive and negative samples turn green and orange, respectively, under LED light at 500-nm wavelength.

#### Confirmatory tests: Internal transcribed spacer 1–polymerase chain reaction and sequencing

**Internal transcribed spacer 1–polymerase chain reaction:** Alongside LAMP, the extracted DNA samples were also analysed with internal transcribed spacer (ITS1)–PCR, which detects both the *Trypanozoon* and other trypanosome parasites and distinguishes the species. The PCR assays were performed in a DNA thermal cycler (Applied Biosystems, California, USA). The ITS1-PCR used two oligonucleotide primers (Table 1) for amplification of ITS1 as described by Njiru et al. (2005). Each amplification reaction was made in a final volume of 20 *μ*L containing 5 *µ*L of 2X Ampdirect plus (Sh imadzu, Japan), 0.5 *µ*M of each oligonucleotide primer for ITS1-PCR, 0.025 U of BIOTAQ HS DNA polymerase (Bioline, UK) and 1 *μ*L of DNA template. The thermocycling programme for the ITS1-PCR was 10 min at 95 °C, followed by 35 cycles of 30 sec at 94 °C, 1 min at 55 °C, 1 min at 72 °C and a final cycle of 7 min at 72 °C. After PCR, 5 *μ*L of each sample was run on a 1.5% agarose gel and stained with GelRed (Biotium), a non-carcinogenic gel staining fluorescence dye.

All samples were also subjected to SRA PCR alongside SRA LAMP to detect *T. b. rhodesiense*. Both forward and reverse primers for SRA PCR were those described by Welburn et al. (2001). A final volume of 20 *μ*L was used for each reaction. The reaction conditions were the same as described above for ITS1-PCR, except the annealing temperature was set as 58 °C.

**Sequencing of internal transcribed spacer 1–polymerase chain reaction products:** Ten samples positive on ITS1-PCR were randomly chosen for sequencing. The PCR products were purified using ExoSap-IT reagents (Affymetrix, USA) as per the manufacturer’s instruction or cloned into pGEMT-easy vector (Promega) if multiple traces were observed. The sequencing reactions were performed using BigDye 3.1 Terminator Cycle sequencing kit and sequenced with an automated 3500 series genetic analyser (Applied Biosystems). The resulting sequences were analysed with the target sequences using MEGA7 software (Sudhir, Stecher & Tamura 2016) and BLAST (https://blast.ncbi.nlm.nih.gov/Blast.cgi).

### Statistical analysis

Data were double entered and verified using Microsoft Excel software. All statistical analyses were performed using R version 3.3.2 (R Core Team 2016).

## Results

A total of 86 mature tsetse flies were captured. All tsetse flies belonged to *Glossina morsitans morsitans* species. Of these tsetse flies, 31 (36%) were male tsetse flies while 55 (64%) were female tsetse flies. All mature tsetse flies were further analysed using dry and liquid RIME LAMP to compare the diagnostic performance of these two tools in detecting *Trypanozoon* trypanosomes in field-caught samples. The results were further confirmed through ITS-PCR and sequencing. We also assessed using SRA PCR and SRA LAMP whether *Trypanozoon*-positive samples were *T. b. rhodesiense* parasites (the causative agent of HAT) or not.

### Detection of *Trypanozoon* trypanosomes using dry and liquid repetitive insertion mobile element loop-mediated isothermal amplification

To evaluate the diagnostic performance of dry RIME LAMP as a potential field surveillance tool, we first used dry RIME LAMP to analyse all 86 field-caught tsetse flies. Dry RIME LAMP detected 14 of 86 (16.3%) samples as *Trypanozoon* positive. Total infected female and male tsetse flies were 12 (14%) and 2 (2.3%), respectively. To verify dry RIME LAMP results, all 86 samples were subjected to a well-established conventional liquid RIME LAMP (Alibu et al. 2015; Malele et al. 2013). Liquid RIME LAMP detected 10 of the 86 (11.6%) samples as *Trypanozoon* positive (Table 2). Furthermore, infected female and male tsetse flies detected using liquid RIME LAMP were 9 (10.5%) and 1 (1.2%), respectively. All liquid RIME LAMP–positive samples were also positive using dry RIME LAMP and the proportion of positive agreement between the two tests was 75% and that for negative agreement was 96%, suggesting that there is no significant difference in the diagnostic performance of dry and liquid RIME LAMP in detecting *Trypanozoon* trypanosomes in field-collected samples.

**TABLE 2:** Diagnostic performance of dry repetitive insertion mobile element loop-mediated isothermal amplification against liquid repetitive insertion mobile element loop-mediated isothermal amplification for the detection of trypanosomes.

Variable	Dry RIME +	Dry RIME −	Total
Liquid RIME +	9	1	10
Liquid RIME −	5	71	76
**Total**	**14**	**72**	**86**

RIME, repetitive insertion mobile element.

### Confirmation of repetitive insertion mobile element loop-mediated isothermal amplification results through internal transcribed spacer 1–polymerase chain reaction

**FIGURE 2:**
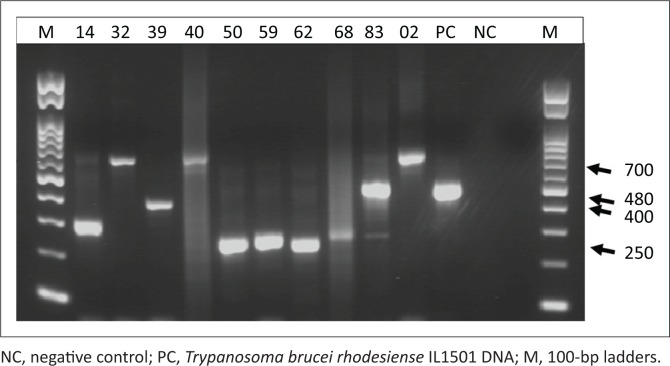
Representative positive products amplified by internal transcribed spacer 1-PCR, polymerase chain reaction were electrophoresed. Sample 14 (300 bp): *Trypanosoma godfleyi*; sample 32, 40 and 02 (710 bp): *Trypanosoma congolense* forest; sample 39 (400 bp): *Trypanosoma simiae*; sample 50, 59, 62, 68 (250 bp): *Trypanosoma vivax*; Sample 83 (480 bp): *Trypanozoon.*

Our group and others have previously described ITS1-PCR as the most specific test for the detection of trypanosomes in tsetse flies (Alibu et al. 2015). Therefore, all 86 tsetse fly samples were reanalysed using ITS1-PCR as a confirmatory test for both dry and liquid RIME LAMP. ITS1-PCR detected 22 of 86 (25.6%) samples as trypanosome positive with trypanosomes infection rate in female and male *G. morsitans* at 30.9% and 12.9%, respectively (*p* = 0.06201, chi-square test) (Table 3). ITS1-PCR showed 5 of 86 (5.8%) of the samples to be *Trypanozoon* positive giving the expected PCR product size of 480 bp (Figure 2) and 17 of 86 (19.8%) samples to be positive for other animal trypanosome species. When a comparison of the results was made, four of five ITS1 *Trypanozoon*-positive samples were also positive using dry and liquid RIME LAMP, hence confirming our LAMP results as a true reflection of *Trypanozoon* infection in field-caught tsetse flies. Thus, in total ITS1-PCR detected 22 of the 86 tsetse flies as infective with trypanosomes. Mixed infection was observed in one of the five *Trypanozoon*-positive samples, indicating that each tsetse fly has the potential of infecting both human and domestic animals (Table 3).

**TABLE 3:** Detection of trypanosome deoxyribonucleic acid by internal transcribed spacer 1-polymerase chain reaction, in *Glossina morsitans morsitans* from Liwonde.

Variable	Female (*n* = 55)		Male (*n* = 31)		Total (*n* = 86)	
	*N*	%	*N*	%	*N*	%
*Trypanozoon*	3/55	5.5	1/31	3.2	4/86	4.7
*Trypanosoma vivax*	4/55	7.3	0/31	0.0	4/86	4.7
*Trypanosoma godfleyi*	3/55	5.5	3/31	9.7	6/86	6.9
*Trypanosoma simiae*	2/55	3.6	0/31	0.0	2/86	2.3
*Trypanosoma congolense*	2/55	3.6	0/31	0.0	2/86	2.3
*T. godfleyi/T. simiae*	2/55	3.6	0/31	0.0	2/86	2.3
*Trypanozoon/T. vivax*	1/55	1.8	0/31	0.0	1/86	1.2
**Total AAT**	**17/55**	**30.9**	**4/31**	**12.9**	**21/86**	**24.4**

AAT, animal African trypanosomiasis.

Because RIME LAMP was developed as a field surveillance tool with high sensitivity (Njiru et al. 2008a), the results of our dry and liquid RIME LAMP when used in field situations are in line with findings of others (Malele et al. 2013). The diagnostic performance of dry RIME LAMP was higher than that of ITS1-PCR (Table 4). Concordance was also observed, and the sensitivity of dry LAMP comparing ITS1-PCR was 80% (95% confidence interval [CI]: 28% – 99%) and specificity was 88% (95% CI: 78% – 94%). Dry RIME LAMP detected 16.3% of the tsetse flies as *Trypanozoon* positive compared to ITS1-PCR, which only detected 5.8% as *Trypanozoon* parasites.

**TABLE 4:** Diagnostic performance of dry repetitive insertion mobile element loop-mediated isothermal amplification against internal transcribed spacer 1-polymerase chain reaction for the detection of trypanosomes.

Variable	ITS1-PCR Trypanozoon+	ITS1-PCR Trypanozoon-	Total
Dry RIME LAMP +	3	11	14
Dry RIME LAMP −	1	71	72
**Total**	**4**	**82**	**86**

ITS1, internal transcribed spacer 1; LAMP, loop-mediated isothermal amplification; PCR, polymerase chain reaction; RIME, repetitive insertion mobile element.

### Confirmation of trypanosome species through sequencing

Because microscopy was not carried out on all field-caught tsetse flies, 10 samples were randomly picked from the 21 clear, single-band–positive ITS1-PCR products which were sequenced and confirmed as Trypanosoma species. The species confirmed were *Trypanozoon* (480 bp), *Trypanosoma vivax* (250 bp), *Trypanosoma godfleyi* (300 bp), *Trypanosoma simiae* (400 bp) and *Trypanosoma congolens*e (7100 bp). Randomly sequenced samples that were also positive on dry and liquid RIME LAMP were all (three samples) confirmed as *Trypanozoon* species. This suggests that dry RIME LAMP can be an alternative surveillance tool because of its user-friendly simplicity compared to ITS1-PCR and liquid RIME LAMP.

### *Trypanosoma brucei rhodesiense* status in tsetse flies using serum-resistant associated gene loop-mediated isothermal amplification and serum-resistant associated gene polymerase chain reaction

Because both dry and liquid RIME LAMP and ITS1-PCR detect all *Trypanozoon* trypanosomes subspecies without distinguishing the subspecies, we next tested if *Trypanozoon*-positive samples detected by RIME LAMP and ITS1-PCR are positive for *T. b. rhodesiense*, which is responsible for human infections in Malawi (Bruce et al. 1914; Chisi et al. 2011; Madanitsa et al. 2009). All *Trypanozoon*-positive samples were subjected to SRA LAMP and SRA PCR as described in the ‘Materials and methods’ section to distinguish *T. b. rhodesiense* from other members of the *Trypanozoon* group. Both SRA LAMP and SRA PCR were negative, suggesting that *Trypanozoon* species detected in RIME LAMP and ITS1-PCR were *T. b. brucei*, a parasite responsible for AAT.

## Discussion

The infection rate of trypanosomes in wild tsetse flies in Malawi was previously shown to be higher compared to other countries in eastern and central Africa where HAT and AAT is endemic (Alibu et al. 2015). In Malawi, the number of new cases of HAT trypanosomiasis infection over the past 10 years have remained constant (Franco et al. 2014) compared to other countries such as Tanzania, Uganda, Zambia and Kenya where the disease is almost under control. This indicates that the disease transmission is not well controlled and humans are still exposed to possibly highly infected vectors and if control is to be possible, surveillance tools should be able to use any available sample materials for the detection of the parasite.

In this study, the infective rate in flies was 25.6% by ITS1-PCR and there was a difference between male (12.9%) and female (30.9%) tsetse flies with higher rates in female *G. m. morsitans*. Unlike in other insect vectors such as sand flies, mosquitoes and black flies (Aransay, Scoulica & Tselentis 2000; Dyab et al. 2015), both male and female tsetse feed on blood and therefore both are vectors for trypanosomes. However, in natural field situations, female tsetse flies usually have higher infection rates than male tsetse flies and this is partly because female tsetse flies have a longer lifespan than male tsetse flies (Leak 1999) and our results were in agreement with this observation. During this study, microscopy was not carried out; however, the ten positive samples on ITS1-PCR that were sequenced confirmed that the samples under study were indeed trypanosomes as they aligned with trypanosome species after BLAST analyses.

Comparative analysis of dry LAMP and liquid LAMP showed that dry LAMP was in concordance with the conventional liquid LAMP where it detected 9 out of 10 positives detected by liquid LAMP. Furthermore, dry LAMP was able to detect five more samples, which were negative on liquid LAMP. This finding is in line with other findings on using LAMP for xenomonitoring of trypanosomes in tsetse flies (Malele et al. 2013).

The ability of ITS1-PCR in detecting *Trypanozoon* subspecies was far less to that of dry RIME LAMP. This is partly because the ITS1 region is estimated to have only 100–200 copies per haploid genome compared to 500 copies in the RIME region, hence making it less sensitive (Bhattacharya, Bakre & Bhattacharya 2002). Therefore, when using PCR for detection of trypanosomes, there is a high possibility of missing out many cases and obtaining misrepresentative data. As shown in Table 3, the performance of dry and liquid RIME LAMP was comparable, detecting 16.3% and 11.6%, respectively. Using dry LAMP as a simple tool for xenomonitoring of trypanosomes in tsetse flies is thus encouraging.

The present study has demonstrated that whole dry dead tsetse flies can be used as samples in both LAMP and conventional PCR. Several other studies have been conducted on the use of tsetse fly mid-guts for LAMP and PCR (Cunningham et al. 2016; Enyaru et al. 2010). The use of whole dead tsetse flies is advantageous for xenomonitoring of trypanosomes in tsetse flies as different species of the trypanosome parasite are found in different parts of the tsetse fly. A good example is *T. vivax*, which only develops in the proboscis of the tsetse fly. This is in contrast to the *T. brucei* group, which develops in the mid-gut of the vector (Leak 1999), hence increasing the possibility of detecting the parasite. There is no need for special storage conditions of the dead flies as they can be stored at room temperature, hence reducing surveillance costs compared to use of mid-guts, which quickly become degraded and require liquid nitrogen tanks to be carried to the field for storage. The dry flies used in this study were left for 3 months at room temperature.

The study has demonstrated robust detection of the dry RIME LAMP method in dead whole tsetse flies. The sensitivity of the dry LAMP was high enough to allow for the detection of a single infected tsetse fly after a long period of storage at room temperature. Though the sample size used in this study was small (86 flies), it still has a potential application for large-scale xenomonitoring for generation of epidemiological data for decision-making. To minimise surveillance costs and save time, use of dry LAMP and dead tsetse flies is recommended over the conventional liquid LAMP and tsetse fly mid-guts.
